# Helminth Community Dynamics in Populations of Blue-Winged Teal (*Anas discors*) Using Two Distinct Migratory Corridors

**DOI:** 10.1155/2011/306257

**Published:** 2011-03-31

**Authors:** Jason M. Garvon, Alan M. Fedynich, Markus J. Peterson, Danny B. Pence

**Affiliations:** ^1^Caesar Kleberg Wildlife Research Institute, Texas A&M University-Kingsville, Kingsville, TX 78363, USA; ^2^Department of Biology, Lake Superior State University, 650 W. Easterday Avenue, Sault Ste. Marie, MI 49783, USA; ^3^Department of Wildlife and Fisheries Sciences, Texas A&M University, College Station, TX 77843, USA; ^4^1616 Blackburn Fork Road, Cookeville, TN 38501, USA

## Abstract

The influence of spatially distinct host subpopulations on helminth community structure and pattern was examined in a migratory avian host species. Forty helminth species represented by 24,082 individuals were collected from 184 blue-winged teal (*Anas discors*; BWT) from 2 primary migratory corridors in Florida (eastern migratory corridor; EMC) and Louisiana and Texas (western migratory corridor; WMC). Mean species richness was greater in BWT from the WMC ($\overset{®}{x}\pm \text{SE}$ = 10.2 ± 0.3 species) than the EMC (8.6 ± 0.2). The helminth community from the WMC had higher abundances of 6 common/intermediate species. Corridor helminth communities were similar in species composition but less similar when incorporating abundances of those species. Overlapping distributions of phylogenetically related host species that share generalist helminth species across ecologically similar habitats seem to mitigate the isolating mechanisms that are necessary for the distinct coevolutionary pathways to develop between adjacent corridors.

## 1. Introduction

Helminth community dynamics among host populations are influenced by several factors such as variations in host feeding ecology [1], habitat use [2], distance between host populations [3–5], the phylogenetic relatedness of host species within an area coupled with host specificity of helminths [6, 7], and the resulting degree of host-parasite coevolution [8]. While many of the above relationships explain differences in helminth community structure (i.e., feeding ecology, habitat use, and distance between populations), others such as phylogenetic relatedness of hosts and host specificity of helminths (generalist rather than specialist) explain observed similarities among helminth communities. 

In addition to the factors mentioned above, helminth communities of waterfowl are subjected to the effects of migration that may accentuate the influence of other community-shaping factors. Several studies concerning helminth communities of waterfowl have addressed loss of helminths between migratory periods [9], effects of temporal variability on helminth communities within a single region [10], and differences in species richness between breeding and wintering grounds [11–13]. These authors tested ecological hypotheses using helminth communities from hosts within a single geographic region or migratory corridor. 

Wallace and Pence [9] proposed that if a migratory host species showed high fidelity to respective migratory corridors, after a substantial period of time, helminth communities unique to hosts within migratory corridors might be formed. Brooks' [8] hypothesis concerning unique forces acting on host-helminth systems within host subpopulations leading to coevolution of hosts and helminths seems to explain the mechanism behind the hypothesis. Thus far, the influence of migratory corridors on the helminth communities using an infracommunity and component community approach within a single host species has not been studied. 

The blue-winged teal (*Anas discors*; BWT) is a migratory waterfowl species that allows for assessment of the degree to which separation of host populations by migratory corridor affects helminth communities, as proposed by Wallace and Pence [9]. Blue-winged teal use and maintain a relatively high rate of fidelity to migratory corridors specific to two primary breeding populations [14], with only 4.8% crossover between corridors reported during fall migration [15]. The eastern migratory corridor (EMC) extends from Manitoba and Minnesota through Florida terminating in the Caribbean and northeastern South America. The western migratory corridor (WMC) extends from Saskatchewan south-southeast to western Louisiana and eastern Texas and terminates in the Yucatan peninsula of Mexico and as far southwest as Peru [14, 16]. Because pairing occurs on the wintering grounds and during spring migration and female birds tend to return to natal sites [16], it is likely that little intermixing, beyond that during fall migration, of populations occurs between corridors. Thus, the BWT populations in each corridor are geographically isolated. Additionally, the far southward migration likely exposes them to helminths not encountered by waterfowl terminating migration in more northern latitudes. 

Our objectives in this study were to examine helminth communities of BWT from two primary migratory corridors, evaluate the degree of similarity in helminth community structure (species richness, prevalence, abundance) and pattern (distribution and diversity), and determine whether or not there are sufficient isolating mechanisms operating on the BWT host-helminth system to allow for coevolutionary processes at the migratory corridor level resulting in two distinct helminth communities.

## 2. Methods

### 2.1. Definitions

Definitions of terms follow Bush et al. [17]. Additionally, common, intermediate, and rare species are those helminths that occurred in >75, 25–75, and <25% of hosts sampled, respectively.

### 2.2. Study Area and Host Collection

Collection of BWT was conducted in accordance with stipulations of the appropriate state and federal permits (FL, WX02177; LA, LNHP-02-107; TX, SPR-0602-222; USF&WS, MB056380-0) and approval by the Texas A&M University-Kingsville Animal Care and Use Committee (Approval number 2002-8-15). Between 21 September and 30 October 2002, as reports from local scouts indicated the presence of migrating BWT, 100 BWT (33 adult male, 20 adult female, 17 juvenile male, 30 juvenile female) were collected from Florida, which represented the EMC, and 84 BWT (18 adult male, 13 adult female, 26 juvenile male, 27 juvenile female) were collected from Louisiana and Texas, which represented the WMC. Collection sites were selected based on availability of legal collection areas and presence of BWT. Equal sample sizes based on sex and age could not be obtained due to limits set by collection permits and because BWT in basic plumage could not be easily sexed prior to collection.

 Within 15 min following death, viscera were removed and fast frozen in ethanol cooled to −70°C with dry ice [18] using procedural modifications outlined by Glass et al. [19]. 

Carcasses were sealed in freezer bags and stored on wet and dry ice with frozen viscera for transport to laboratory freezers where they were stored at −10°C until necropsy.

### 2.3. Helminth Collection

Upon necropsy, viscera were divided into the following microhabitats: (T) trachea and larynx (when present), (L) lung, (H) heart, (LV) liver and gallbladder, (E) esophagus, (P) proventriculus, (G) gizzard, (PA) pancreas, (SI) small intestine, (LI) large intestine, (CL) cloaca, (C) ceca, (ME) mesenteric veins, kidney and spleen, (B) bursa (when present) and female reproductive tract, and (MW) miscellaneous wash (rinse from the freezer bag). The following microhabitats from carcasses also were examined for helminths: (EY) eye, (BI) bill, (SN) nasal sinus, suborbital sinus and nasal cavity, and (S) subcutaneous tissue. 

Nematodes were fixed in glacial acetic acid and stored in 70% ethanol with 8% glycerin. Acanthocephalans, cestodes, and trematodes were preserved in acetic acid-formalin-ethanol (AFA) and stored in 70% ethanol. Nematodes were identified in alcohol-glycerin wet mounts. Acanthocephalans, cestodes, and trematodes were identified in wet mounts, if possible. Otherwise, they were stained using Semichon's acetocarmine and mounted in Canada balsam permanent mounts. Identification of helminths was based solely on morphological characteristics and followed the taxonomic keys of McDonald [20–22] and Czaplinski and Vaucher [23]. Representative samples were deposited in the U.S. National Parasite Collection, Beltsville, MD 20705, U.S.A. (USNPC nos. 103158-103195).

### 2.4. Statistical Analyses

Statistical analyses were performed using SPSS version 11.0. Reported differences are significant at *P* ≤ .05. Infracommunity and component community abundance values from the common/intermediate species were not normally distributed as determined by the Shapiro-Wilk statistic (W) and, therefore, were rank transformed [24] prior to further analyses. Abundance transformations of each species were performed independently to avoid potential problems associated with dissimilar distribution patterns [25].

Variation in prevalence of common/intermediate species among migratory corridor, host age, and host sex as well as age and sex within migratory corridors was analyzed using *χ* ^2^ [26]. Differences in species richness among hosts were analyzed by Analysis of Variance (ANOVA) to determine the influence of extrinsic variables (migratory corridor) and intrinsic variables (host age and host sex) on the overall helminth community and helminth communities within corridors (intrinsic only). 

The influence of main and interactive effects of extrinsic and intrinsic variables on individual and combined common/intermediate species rank abundances was assessed using ANOVA and Multivariate Analysis of Variance (MANOVA) with an associated *F* statistic and Wilk's lambda (Λ), respectively, [10, 27]. If significant interactions were detected for ANOVA or MANOVA, main effects were not considered and pairwise comparisons were used to identify points of interaction.

The numerical dominance index (DI) of Leong and Holmes [28] was calculated for the collective host population and for the respective intrinsic and extrinsic variables. The sum of the minimum number of DI values needed to account for 75% of helminth individuals in a particular community (ND_75_) was created and calculated to aid in interpretation of numerical dominance within helminth communities. The ND_75_ values are presented as ND_75_ [*a*, *b*], where *a* is the minimum number of DI values needed to equal or exceed 75 and *b* is the total number of species present in the respective community. 

 Differentiation diversity (*β*) was measured using two similarity measures for the main effects variables of corridor, host age and host sex plus host age, and sex within corridors. The percent similarity index (PS; [29]) was used to evaluate the similarity of helminth species abundances between component communities based on the relative proportion of all helminth individuals contributed by each helminth species [30]. Similarity of shared helminth species was evaluated using the coefficient of community index (CC) of Krebs [29].

## 3. Results

The overall component community contained 24,082 helminth individuals representing 40 species (1 acanthocephalan, 9 cestodes, 10 nematodes, and 20 trematodes). Fifteen species (*Corynosoma constrictum*,* Cloacotaenia megalops*,* Echinocotyle rosseteri*,* Gastrotaenia cygni*,* Amidostomum acutum*,* Capillaria anatis*,* Capillaria contorta*,* Epomidiostomum uncinatum*,* Streptocara crassicauda*,* Tetrameres ryjikovi*,* Apatemon gracilis*,* Dendritobilharzia pulverulenta*,* Echinoparyphium recurvatum*,* Echinostoma trivolvis*,* and Trichobilharzia querquedulae*) were considered common/intermediate (>25% prevalence overall) and accounted for 79.3% of all helminth individuals collected. 

The component communities from both the EMC and WMC contained 36 species (Table 1). The component community from juvenile BWT had 2 more species (37) than that of adult BWT (35), and the component communities from males and females each had 37 species.

### 3.1. Infracommunity Analyses

Blue-winged teal were infected with 3–17 helminth species and averaged 9.3 ± 0.2 ($\overset{®}{x}\pm \text{SE}$) species. Infracommunities of BWT from the EMC had lower mean species richness (8.6 ± 0.2 species, range 3–15 species) than those in the WMC (10.2 ± 0.3, range 5–17). Within the collective host sample, a significant age by sex interaction did not allow for the effects of age and sex alone to be determined. Infracommunity species richness from juvenile male BWT (11.2 ± 0.4, range 5–17) was higher than adult males (8.0 ± 0.4, range 3–14), adult females (8.7 ± 0.4, range 4–14), and juvenile females (9.3 ± 0.3, range 5–15). Juvenile females also had higher infracommunity species richness than adult males. 

Within the EMC, a significant age by sex interaction was observed. Species richness in juvenile males (10.4 ± 0.6, range 6–14) was higher than that of adult males (7.4 ± 0.4, range 3–11), adult females (8.6 ± 0.4, range 4–12), and juvenile females (9.0 ± 0.4, range 5–15). 

Within the WMC, infracommunity species richness was lower in adults (9.0 ± 0.5, range 4–15) than juveniles (10.6 ± 0.4, range 5–17). Species richness between males (10.6 ± 0.4, range 5–17) and females (9.4 ± 0.4, range 5–15) was not significantly different.

### 3.2. Component Community Analyses

Helminth communities of BWT from the WMC had higher prevalence of 7 species, while 2 were more prevalent in communities from the EMC (Table 2). Prevalence values of 5 species were higher in juveniles than adults while *C. megalops *and *T. querquedulae *were higher in adults. By sex, prevalence of 2 species was higher in males than females. 

 Distribution patterns of abundance of the 15 common/intermediate species were nonnormally distributed overall and for each of the main effects of migratory corridor, host age, and host sex. Rank abundances of the 15 common/intermediate species varied by extrinsic and intrinsic factors across the host sample and varied by intrinsic factors within corridors. Seven species differed in mean ranked abundance between corridors, and only *D. pulverulenta* was higher in the EMC (Table 3). By host age, *T. querquedulae* was the only one of 6 species with abundances that were significantly higher in adults, and only *E. trivolvis *abundances differed across sexes (Table 3).

 Within the EMC, ranked abundance of *C. anatis* was higher in adults, while abundances of *E. uncinatum *and *T. ryjikovi* were higher in juveniles. There was a significant age by sex interaction for *C. megalops*, where mean ranked abundance was higher in adult females than juvenile males. Within the WMC, juveniles had significantly higher mean ranked abundances of *C. constrictum*, *E. uncinatum*, *A. gracilis*, and *E. recurvatum*, while adults had higher mean ranked abundances of *C. megalops *and *T. querquedulae*. Males had significantly higher mean ranked abundance of *E. trivolvis *than females. 

 Collectively, mean ranked abundances of the 15 common/intermediate helminths were greater in the WMC than the EMC, and in adults than juveniles. Within corridors, the collective ranked abundance of common/intermediate helminths was greater in adults from the EMC than juveniles and from the WMC was higher in juveniles than adults and in males than females.

#### 3.2.1. Numerical Dominance and Community Similarity Analyses

Of the 40 helminth species collected, *T. quequedulae* numerically dominated (DI = 34.1) the overall component community. Only 14 of the remaining 39 species accounted for >1% of all individuals; 4 of these, *Pseudospelotrema* sp. (7.7%), *P. oxyurus* (4.6%), *M. pygmaeus* (3.2%), and *S. filumferens* (1.2%) rarely occurred (Table 4). The overall community had an ND_75_ [7, 40] indicating that 7 of 40 species found represented over 75% of all individual helminths in that community. 

*Pseudospelotrema *sp. accounted for 12.9% of individuals from the EMC and was the only other species representing >10% of all individual helminths within BWT from any community delineated by extrinsic or intrinsic factors. The ND_75_ values were similar for helminth communities between corridors and the same age classes between corridors; they varied between age classes overall and within each corridor (Table 4). Overall, helminth communities from juveniles had higher ND_75_ values than adults and were less dominated by *T. querquedulae*. 

Among those communities with similar ND_75_, species comprising those values varied among corridors and age classes. For example, while juveniles from the EMC had a similar ND_75_ [7, 34] value to that of the WMC [7, 36], only four species (*T. querquedulae*, *E. rosseteri*, *E. recurvatum*, and *A. acutum*) were represented in both groups (Table 4). Similarly, only 2 species (*T. querquedulae* and *E. rosseteri*) were represented in both adult groups from the EMC and WMC. 

Helminth communities from BWT in the 2 corridors and those of juvenile and adult BWT were similar in species composition. However, the latter were less numerically similar than the former based on PS and CC values (Table 5). Helminth communities from adult and juvenile BWT in the WMC shared more species than other communities yet were nearly the lowest in numerical similarity. Communities from adults and juveniles in the EMC were most similar in PS and CC values.

## 4. Discussion

The helminth communities of BWT across the EMC and WMC in North America showed many similarities in both structure and pattern. The BWT using both migratory corridors had an equal degree of species richness, sharing all the common/intermediate and most of the rare species. Additionally, all of the common/intermediate helminths were waterfowl generalists rather than specialists of any one species. This indicates that host-sharing of helminths coinfecting phylogenetically similar species may be the largest factor affecting the overall component community composition and that complete geographic isolation has not occurred in the BWT host-helminth system. However, there were differences in infracommunity species richness and relative abundances of some rare but numerically dominant helminths from hosts between corridors. This suggests that certain isolating mechanisms such as relative abundance and differential habitat use of these BWT populations as well as those of phylogenetically related hosts influenced helminth community composition. 

Similarity in helminth community composition likely occurred due to BWT sharing habitat with related waterfowl species. There are 10 species of the genus *Anas* in North America [14], all of which may have some degree of overlapping habitat utilization with BWT at some time during the year. Additionally, McDonald [31] considered 13 of the 15 common/intermediate species from the present study characteristic or commonly occurring species of the family Anatidae, which has cosmopolitan distribution throughout the world. Since McDonald's [31] report, *C. constrictum* has been described as a characteristic waterfowl parasite [22] and *T. querquedulae* has been found to occur more frequently [9, 32] than previously indicated. Similarity of habitats and intermediate invertebrate hosts in core nesting regions across the two migratory corridors diminishes the likelihood of divergence in host helminth community structure and composition. Potentially, it is also important to remember that similarity and/or dissimilarity in environmental characteristics and invertebrate populations across these extensive geographic areas could be dramatically affected by short- or long-term changes in weather and climatic patterns. We made no attempt to monitor or analyze weather patterns across these migratory corridors, other than casually noting the absence of any unusual or radical changes in temperature and precipitation across the regions either immediately preceding or during the collecting period of this study. 

Observed divergence in helminth communities between migratory corridors also can be explained by host sharing of helminths since the degree of host sharing may have been different between migratory corridors based on relative abundance and phylogenetic relatedness of BWT to other host species. There is greater species richness and population density of the collective waterfowl species that comprise the WMC compared to those that share the EMC (see [14, page 22]). These could be facilitating density-dependent factors in helminth transmission for the western Anatini. We found that WMC helminth communities showed higher mean numbers of species per host and higher mean abundances in 6 of 7 helminth species that differed significantly between corridors. The exception was a higher mean abundance of *D. pulverulenta* in EMC BWT, but this helminth is considered an accidental helminth of dabbling ducks and normally infects diving ducks (tribe Aythyini) and mergansers (tribe Mergini) [33]. Additionally, *Pseudospelotrema *sp., which normally infects plovers, herons and seaside sparrows (*Ammospiza maritrima*) [34, 35], accounted for a high proportion of all individual helminths. The high abundances of *Pseudospelotrema *sp. and two other rare saltwater species likely led to the observed difference in ranked abundances of the collective common/intermediate species between corridors. 

Differential use of habitat types and other differences in host ecology of the respective waterfowl populations may have been important factors in determining helminth community structure across the two migratory corridors. Although helminth communities between migratory corridors were similar in species composition (CC = 86.5), they were less similar in numerical composition (PS = 59.3) and those species representing the ND_75_ values. Most prominent was the numerical dominance of the EMC community by 3 saltwater trematode species (*M. pygmaeus, P. oxyurus,* and *Pseudospelotrema* sp.) (Table 4) that were rarely found in the WMC and may infect a variety of other host species. Bush [36] found that helminth communities of willets (*Tringa semipalmata*) feeding in both fresh and saltwater were different from those that fed exclusively in freshwater. Similarly, Fedynich et al. [4] noted richer helminth communities in mottled ducks using multiple versus single habitats. Likewise, Fedynich et al. [2] found differences in helminth communities of two whistling duck species that had different feeding strategies, thereby altering exposure probabilities to helminth infective stages. We found that the combined numbers of individuals of three helminth species (*C. constrictum*, *S. crassicauda*, and *T. ryjikovi*) accounted for 17.3% more of the helminth community in the WMC versus EMC. These species require a freshwater amphipod intermediate host [31] that is most common in clear unpolluted waters in North America [37]. We believe that habitat availability and host habitat use and/or foraging behavior likely influenced these helminth communities.

### 4.1. Host Age and Sex

Variation in prevalence and mean abundance of helminths based on intrinsic host factors followed common trends in parasite-host relationships, although a few interesting trends arose. Comparisons of those species comprising the ND_75_, by host age class between migratory corridors illustrated how isolating mechanisms may be operating. Two characteristic BWT helminth species (*T. querquedulae* and *E. rosseteri*) were present in all ND_75_ communities. The remaining ND_75_ species in adult BWT were corridor specific. These and two other species (*A. acutum* and *E. recurvatum*) were dominant in EMC and WMC juvenile communities. Similarity of dominant helminths in juveniles indicates a characteristic suite of parasites encountered throughout the breeding grounds. Alternatively, uniqueness of the adult communities indicates corridor-specific divergence due to differences in wintering grounds habitat or differential host sharing of helminths.

As expected from previous studies [9, 10, 38, 39], we found that mean prevalence or abundance values of most (5 of 7 species) common/intermediate helminth species were highest in juvenile versus adult BWT. The precocial nature of waterfowl increases diet breadth as compared to altricial young of other birds combined with the high proportion of invertebrates ingested by ducklings [40] to meet their high protein demands for growth [41] which has been suggested as a cause of higher exposure rates to helminth infective stages that use invertebrate intermediate hosts [42]. Additionally, juveniles may initially lack immunity to infectious agents for the first few weeks of life [36], which temporarily allows increased species richness and intensity of infections. 

 Observed higher-ranked abundance of the collective common/intermediate helminths in adult BWT was likely due to the large numbers of *T. querquedulae* and to the rarely occurring saltwater trematodes of which 3 species represented 1/3 to 4 times more of the helminth community in adult than juvenile birds. Certain of these infections may represent cumulative exposures to immunologically resistant tissue dwelling helminths as evident by the 100% prevalence of *T. querquedulae*. Heavy infections of adult BWT with saltwater trematodes may represent delayed encounters of just matured but still immunologically naive adult birds. Saltwater trematodes could be acquired during their first migration into saltwater areas from their freshwater nesting areas.

 Helminth communities from male BWT displayed higher prevalence and both prevalence and abundance of *D. pulverulenta* and *E. trivolvis*, respectively. The normal definitive hosts of *D. pulverulenta* are diving ducks and mergansers, [33]. Male BWT spend the molt on open water [16] where these ducks are more likely to occur. Variation in habitat use may also explain trends in abundance of *E. trivolvis*. Fedynich et al. [30] attributed the higher prevalence of *T. tenuis* in male white-fronted geese (*Anser albifrons*) to differential habitat use and feeding patterns between nesting, nonbreeding, and molting individuals. 

 In summary, the use of 2 migratory corridors by BWT has not been a sufficient isolating mechanism to result in a completely unique helminth community structure and pattern within each corridor. Most likely a combination of host sharing by helminths and overlapping distributions of many phylogenetically similar host species across migratory corridors of BWT has prevented the formation of 2 separate host-helminth systems. However, variation in numerical dominance of helminth species within host communities was observed between migratory corridors, likely resulting from differences in relative abundance and phylogenetic relatedness of hosts shared by helminths, habitats used by hosts within the corridors, and diet within corridors. Assessment of numerical dominance and the newly created ND_75_ showed promise in illustrating the shift from characteristic suites of helminths in juveniles from both corridors to the uniqueness of helminth communities in adults from each corridor. 

Assessment of helminth community structure is a useful tool for examining the extent to which host geographic separation influences helminth populations if a large degree of isolation has occurred over a long period of time. However, there are other methods for examining more recent isolation events. In addition to focusing on species composition, future studies addressing helminth community isolation in migratory hosts should also incorporate analysis of microsatellite or internal transcribed spacer (ITS) regions of ribosomal RNA from both host and helminth populations in order to determine if they truly are isolated. Also, the influence of weather and climatic patterns needs to be considered, as long-term shifts in regional weather may influence intermediate host availability.

## Figures and Tables

**Table 1:** Number of hosts infected (*n*), prevalence (%), and mean abundance ($\overset{®}{x}\pm \text{SE}$) of 40 helminth species from 184 blue-winged teal (*Anas discors*) collected from eastern and western migratory corridors.

	Prevalence	Abundance		Eastern corridor		Western corridor	
	*n* (%)	$\overset{®}{x}\pm \text{SE}$	Total	*n* (%)	Abundance $\overset{®}{x}\pm \text{SE}$	*n* (%)	Abundance $\overset{®}{x}\pm \text{SE}$
Acanthocephala							
* Corynosoma constrictum*, SI, LI	101 (54.9)	8.4 ± 1.5	1,553	37 (37.0)	1.9 ± 0.6	64 (76.2)	16.2 ± 2.9

Cestoda							
* Cloacotaenia megalops*, CL	146 (79.3)	4.7 ± 0.4	870	80 (80.0)	4.1 ± 0.4	67 (79.8)	5.4 ± 0.7
*Diorchis* sp., SI, LI	26 (14.1)	0.7 ± 0.2	129	13 (13.0)	0.9 ± 0.4	13 (15.5)	0.7 ± 0.1
*Drepanidotaenia* sp., L, MW	7 (3.8)	0.2 ± 0.1	29	1 (1.0)	0.1 ± 0.1	6 (7.1)	0.3 ± 0.1
* Echinocotyle rosseteri*, SI, LI	115 (62.5)	12.1 ± 2.7	2,220	61 (61.0)	8.7 ± 2.2	54 (64.3)	16.1 ± 5.2
Hymenolepis sp*, SI	6 (3.2)	0.5 ± 0.2	87	2 (2.0)	0.4 ± 0.4	4 (4.8)	0.6 ± 0.5
* Fimbraria fasciolaris*, SI, LI	14 (7.6)	0.2 ± 0.1	30	10 (10.0)	0.3 ± 0.1	4 (4.8)	0.1 ± <0.1
* Gastrotaenia cygni*, G	49 (26.6)	0.7 ± 0.1	137	19 (19.0)	0.6 ± 0.1	30 (35.7)	1.0 ± 0.2
*Microsomacanthus hopkinsi*, SI, LI	13 (7.1)	0.9 ± 0.4	162	7 (7.0)	0.6 ± 0.3	6 (7.1)	1.3 ± 0.8
*Sobolevicanthus filumferens*, SI, LI	22 (12.0)	1.5 ± 0.6	284	3 (3.0)	0.8 ± 0.7	19 (22.6)	2.5 ± 0.1

Nematoda							
*Acuraria* sp. (larvae), E	6 (3.3)	0.1 ± <0.1	14	—	—	6 (7.1)	0.2 ± 0.1
* Amidostomum acutum*, G	169 (91.8)	6.9 ± 0.4	1,276	96 (96.0)	6.9 ± 0.5	73 (86.9)	6.9 ± 0.7
*Capillaria anatis*, C	69 (37.5)	1.0 ± 0.1	179	28 (28.0)	0.6 ± 0.2	41 (48.8)	1.4 ± 0.2
*Capillaria contorta*, BI, E	84 (45.7)	1.3 ± 0.2	238	48 (48.0)	1.2 ± 0.2	36 (42.9)	1.4 ± 0.4
*Epomidiostomum uncinatum*, G	49 (26.6)	0.9 ± 0.2	163	30 (30.0)	0.9 ± 0.2	19 (22.6)	0.9 ± 0.2
* Porrocaecum crassum*, SI^A, M^	1 (0.5)	<0.1	2	1 (1.0)	<0.1 ± <0.1	—	—
*Sacronema* sp., S	6 (3.3)	<0.1 ± <0.1	8	4 (4.0)	0.1 ± <0.1	2 (2.4)	<0.1 ± <0.1
* Streptocara crassicauda*, G	118 (64.1)	5.0 ± 0.7	919	56 (56.0)	2.4 ± 0.4	62 (73.8)	8.1 ± 1.3
*Tetrameres ryjikovi*, P	89 (48.4)	2.4 ± 0.3	448	38 (38.0)	1.7 ± 0.4	51 (60.7)	3.4 ± 0.6
* Tetrameres striata*, P^A, F^	1 (0.5)	<0.1	3	1 (1.0)	<0.1 ± <0.1	—	—

Digenea							
* Apatemon gracilis*, SI, LI	76 (40.3)	7.0 ± 1.8	1,290	24 (24.0)	1.6 ± 0.5	52 (61.9)	13.5 ± 3.8
*Dendritobilharzia pulverulenta*, H	50 (27.2)	0.4 ± 0.1	73	34 (34.0)	0.5 ± 0.1	16 (19.1)	0.3 ± 0.1
*Echinoparyphium recurvatum*, SI, LI	52 (38.3)	6.7 ± 1.8	1,237	19 (19.0)	7.5 ± 3.1	35 (41.7)	5.8 ± 1.6
*Echinostoma trivolvis*, SI, LI	70 (38.0)	1.6 ± 0.3	296	41 (41.0)	2.0 ± 0.4	31 (36.9)	1.2 ± 0.3
* Eucotyle zakharowi*, SI, LI, C	6 (3.3)	0.1 ± 0.0	11	2 (2.0)	<0.1 ± <0.1	4 (4.8)	0.1 ± 0.1
*Hyptiasmus arcuatus*, SN	22 (12.0)	0.2 ± 0.1	39	6 (6.0)	0.1 ± <0.1	17 (20.2)	0.4 ± 0.1
* Microphallus pygmaeus*, C	16 (8.7)	4.2 ± 1.6	776	14 (14.0)	7.2 ± 0.3	2 (2.4)	0.7 ± 0.5
*Notocotylus attenuatus*, C	16 (8.7)	1.1 ± 0.5	196	3 (3.0)	0.3 ± 0.2	13 (15.5)	2.1 ± 1.1
* Notocotylus breviserialas*, C^J, F^	4 (2.2)	0.4 ± 0.3	71	4 (4.0)	0.7 + 0.5	—	—
*Paramonostomum alveatum*, C^J, F^	2 (1.1)	0.0 ± 0.0	2	2 (2.0)	<0.1 ± <0.1	—	—
*Philophthalmus* sp., EY, SN	11 (6.0)	0.3 ± 0.2	50	9 (9.0)	0.5 ± 0.3	2 (2.4)	<0.1 ± <0.1
* Prosthogonimus cuneatus*, B^J^	11 (6.0)	0.2 ± 0.1	31	8 (8.0)	0.2 ± 0.1	4 (4.8)	0.1 ± <0.1
*Pseudospelotrema* sp., C	34 (18.5)	10.1 ± 3.1	1,864	26 (26.0)	17.5 ± 5.6	8 (9.5)	1.5 ± 0.1
* Psilochasmus oxyurus*, SI, LI	32 (17.4)	6.0 ± 1.8	1,104	29 (29.0)	11.0 ± 3.4	4 (4.8)	0.1 ± <0.1
Schistosome sp., MW^A, M^	1 (0.5)	<0.1	1	—	—	1 (1.2)	<0.1 ± <0.1
*Strigea* sp., SI^J, M^	1 (0.5)	<0.1	4	—	—	1 (1.2)	<0.1 ± <0.1
* Trichobilharzia querquedula*, LV, PA, ME	175 (95.1)	44.5 ± 4.4	8,193	97 (97.0)	53.0 ± 7.2	78 (92.9)	34.4 ± 4.1
*Typhlocoelum sisowi*, T	26 (14.1)	0.3 ± 0.1	57	8 (8.0)	0.1 ± <0.1	18 (21.4)	0.5 ± 0.1
*Typhlophilus* sp., SI^J^	3 (1.6)	0.1 ± 0.1	26	—	—	2 (2.4)	0.3 ± 0.3
* Zygocotyle lunata*, C	7 (3.8)	<0.1 ± <0.1	8	3 (3.0)	<0.1 ± <0.1	4 (4.8)	0.1 ± <0.1

Total	184 (100)	103.7 ± 6.2	24,082	100 (100)	93.4 ± 8.7	84 (100)	116.1 ± 8.6

B: bursa and female reproductive tract, BI: bill, C: ceca, CL: cloaca, E: esophagus, EY: eye, G: gizzard, H: heart, L: lung, LI: large intestine, LV: liver and gallbladder, ME: mesenteric veins, kidney and spleen, MW: miscellaneous wash, P: proventriculus, PA: pancreas, S: subcutaneous tissue, SI: small intestine, SN: nasal sinus, suborbital sinus and nasal cavity, T: trachea and larynx.

*Specimens damaged or immature cestodes that could not be identified beyond family.

^A^Species found only in adults, ^J^Species found in only in juveniles.

^M^Species found only in males, ^F^Species found only in females.

**Table 2:** Prevalence of the 15 common/intermediate species by migratory corridor, host age, and host sex from 184 blue-winged teal collected in Florida, Louisiana, and Texas from 21 September through 30 October 2002.

	Corridor		Age		Sex	
	East	West	Adult	Juvenile	Male	Female
Species						
* Corynosoma constrictum*	37.0	**76.2**	32.1	**74.0**	50.0	60.0
*Cloacotaenia megalops *	80.0	78.5	**88.1**	72.0	77.7	81.1
*Echinocotyle rosseteri *	61.0	64.3	58.3	66.0	60.6	64.4
*Gastrotaenia cygni *	19.0	**35.7**	32.1	22.0	30.9	22.2
*Amidostomum acutum *	**96.0**	86.9	91.6	92.0	93.6	90.0
*Capillaria anatis *	28.0	**48.8**	23.8	**49.0**	36.2	38.8
*Capillaria contorta *	48.0	42.9	40.5	50.0	48.9	42.2
*Epomidiostomum uncinatum *	**30.0**	22.6	13.1	38.0	25.5	27.7
*Streptocara crassicauda *	56.0	**73.8**	64.2	64.0	61.7	66.6
*Tetrameres ryjikovi *	38.0	**60.7**	34.5	**60.0**	43.6	53.3
*Apatemon gracilis *	24.0	**61.9**	33.3	**48.0**	46.8	35.5
*Dendritobilharzia pulverulenta *	34.0	19.0	**33.3**	22.0	**34.0**	20.0
*Echinoparyphium recurvatum *	19.0	**39.3**	20.2	**35.0**	29.7	26.6
*Echinostoma revolutum *	41.0	34.5	35.7	40.0	**45.7**	30.0
*Trichobilharzia querquedulae *	97.0	92.8	**100.0**	91.0	95.7	94.4

*Numbers in** Bold** denote Significant difference (*P* ≤ .05) in prevalence.

**Table 3:** Abundance values ($\overset{®}{x}\pm \text{SE}$) for the 15 common/intermediate by host migratory corridor, host age, and host sex for 184 blue-winged teal collected in Florida, Louisiana, and Texas from 21 September through 30 October 2002.

	Corridor		Age		Sex	
	East	West	Adult	Juvenile	Male	Female
Species						
* Corynosoma constrictum*	1.9 ± 0.6	**16.2** **±** **2.9**	3.4 ± 1.5	**12.7** **±** **2.3**	9.2 ± 2.5	7.7 ± 1.4
*Cloacotaenia megalops *	4.1 ± 0.4	5.5 ± 0.7	5.4 ± 0.5	4.2 ± 0.6	4.8 ± 0.6	4.6 ± 0.5
*Echinocotyle rosseteri *	8.7 ± 2.2	16.1 ± 5.2	13.3 ± 5.2	11.0 ± 2.2	9.2 ± 2.1	15.0 ± 5.0
*Gastrotaenia cygni *	0.6 ± 0.1	**1.0** **±** **0.2**	0.9 ± 0.2	0.6 ± 0.2	0.9 ± 0.2	0.5 ± 0.1
*Amidostomum acutum *	6.9 ± 0.5	6.9 ± 0.7	6.8 ± 0.6	7.1 ± 0.6	7.0 ± 0.6	6.8 ± 0.6
*Capillaria anatis *	0.6 ± 0.2	**1.4** **±** **0.2**	0.7 ± 0.2	1.2 ± 0.2	1.2 ± 0.3	0.8 ± 0.1
*Capillaria contorta *	1.2 ± 0.2	1.4 ± 0.4	1.0 ± 0.2	1.5 ± 0.4	1.3 ± 0.2	1.3 ± 0.4
*Epomidiostomum uncinatum *	0.9 ± 0.2	0.9 ± 0.2	0.2 ± 0.1	**1.5** **±** **0.3**	1.0 ± 0.2	0.7 ± 0.2
*Streptocara crassicauda *	2.4 ± 0.4	**8.1** **±** **1.3**	2.4 ± 0.3	**7.2** **±** **1.1**	4.4 ± 0.8	5.6 ± 1.0
*Tetrameres ryjikovi *	1.7 ± 0.4	**3.4** **±** **0.6**	1.5 ± 0.4	**3.2** **±** **0.5**	1.9 ± 0.4	3.0 ± 0.6
*Apatemon gracilis *	1.6 ± 0.5	**13.5** **±** **3.8**	1.8 ± 0.8	**11.4** **±** **3.2**	7.6 ± 2.8	6.4 ± 2.3
*Dendritobilharzia pulverulenta *	**0.5** **±** **0.1**	0.3 ± 0.1	0.5 ± 0.1	0.4 ± 0.1	0.4 ± 0.1	0.4 ± 0.1
*Echinoparyphium trivolvis *	7.5 ± 3.1	5.8 ± 1.6	4.9 ± 1.9	8.3 ± 3.0	**8.1** **±** **3.2**	5.2 ± 1.8
*Echinostoma revolutum *	2.0 ± 0.4	1.2 ± 0.3	1.1 ± 0.2	2.0 ± 0.5	2.1 ± 0.5	1.1 ± 0.3
*Trichobilharzia querquedulae *	53.0 ± 7.2	34.4 ± 4.1	**61.4** **±** **8.3**	30.4 ± 3.6	50.4 ± 7.7	38.4 ± 3.8

*Numbers in** Bold** denote Significant difference (*P* ≤ .05) in ranked abundance.

**Table 4:** Numerical dominance values of species accounting for <1% of all helminths, and ND_75_ values, for the overall helminth community of blue-winged teal (*Anas discors*) and those delineated by host migratory corridor, age, and age within each migratory corridor.

		Corridor		Age		Eastern corridor		Western corridor	
	Overall	East	West	Adult	Juvenile	Adult	Juvenile	Adult	Juvenile
ND_75_	[7, 40]	[6, 36]	[7, 36]	[5, 36]	[9, 39]	[4, 29]*	[7, 34]	[5, 33]	[7, 36]
Species									
* T. querquedulae*	**34.07**	**39.63**	**27.06**	**46.48**	**23.52**	**47.63**	**30.01**	**44.28**	**17.46**
* E. rosseteri*	**9.20**	**6.44**	**12.68**	**10.06**	**8.48**	**6.60**	**6.29**	**16.61**	**10.48**
* Pseudospelotrema* sp.	**7.72**	**12.94**	1.14	**9.32**	**6.36**	**12.78**	**13.28**	2.74	0.25
* C. constrictum*	**6.43**	1.42	**12.74**	2.54	**9.74**	0.28	2.79	**6.84**	**16.03**
* A. gracilis*	**5.34**	1.15	**10.62**	1.35	**8.74**	0.44	2.01	3.08	**14.82**
* A. acutum*	**5.33**	5.24	**5.45**	**5.12**	**5.52**	5.12	**5.24**	**5.12**	**5.64**
* E. recurvatum*	**5.12**	**5.27**	**4.57**	3.70	**6.33**	4.92	**6.39**	1.38	**6.34**
* P. oxyurus*	4.57	**8.15**	0.05	3.56	**5.43**	**6.60**	**6.29**	0.03	0.06
* S. crassicauda*	3.81	1.78	**6.36**	1.79	**5.52**	1.44	2.19	2.46	**8.54**
* C. megalops*	3.63	3.11	**4.28**	4.07	3.25	3.27	2.84	**5.59**	**3.56**
* M. pygmaeus*	3.21	**5.31**	0.57	**5.19**	1.53	**7.08**	**3.27**	1.59	0.00
* T. ryjikovi*	1.87	1.25	2.65	1.14	2.49	0.63	1.94	2.09	2.96
* E. revolutum*	1.23	1.36	0.93	0.83	1.56	0.96	2.07	0.57	1.12
* S. filumferens*	1.18	0.56	1.95	0.40	1.84	0.00	1.24	1.15	2.39
* C. contorta*	1.00	0.91	1.10	0.76	1.20	0.74	1.08	0.78	1.28

**Bold** numbers denote those values used to calculate ND_75_ values

*Two species have equal values that could be used to calculate ND_75_.

**Table 5:** Percent Similarity (PS) and Coefficient of Community (CC) values for helminth communities of blue-winged teal (*Anas discors*) by host migratory corridor, host age, host sex, and age and sex within corridors.

Level of comparison	PS^a^	CC^a^
Corridor	59.3	86.5
Age	70.0	85.3
Sex	83.1	92.1
East (Age)	77.0	81.3
East (Sex)	76.0	86.2
West (Age)	59.5	90.0
West (Sex)	82.5	89.6

^a^Percent Similarity (PS) and Coefficient of Community (CC) values range from 0 (completely dissimilar) to 100 (completely similar).
